# Supplementary material for: Assessing RNA-Seq Workflow Methodologies Using Shannon Entropy
Source: Biology (Basel). 2024 Jun 28;13(7):482. doi: 10.3390/biology13070482 (PMC11274087; doi:10.3390/biology13070482)
Supplement: Supplementary file 1 [file biology-13-00482-s001.zip › biology-3035206-supplementary/Figure S1.pdf]

A: BLCA

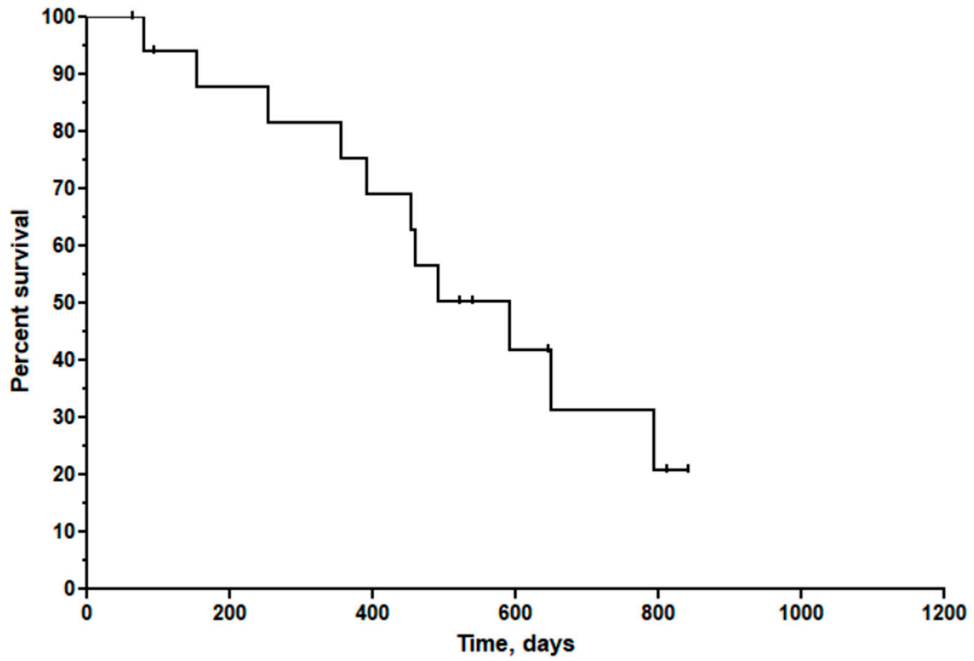
Percent survival
100
90
80
70
60
50
40
30
20
10
0
0
200
400
600
800
1000
1200
Time, days

B: COAD

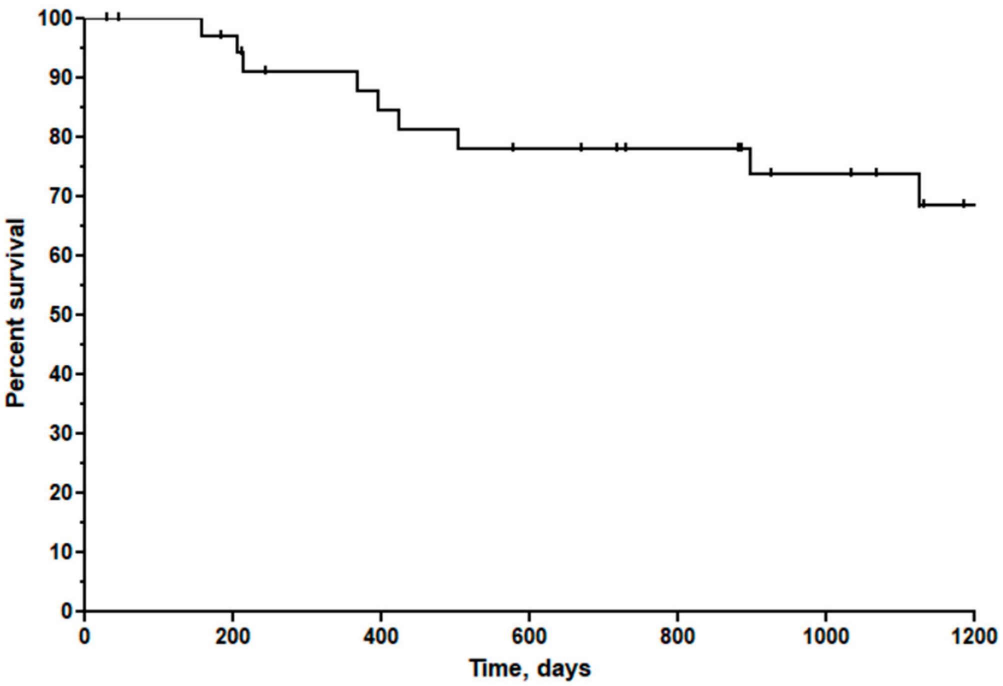
Percent survival
100
90
80
70
60
50
40
30
20
10
0
0
200
400
600
800
1000
1200
Time, days

C: UCS

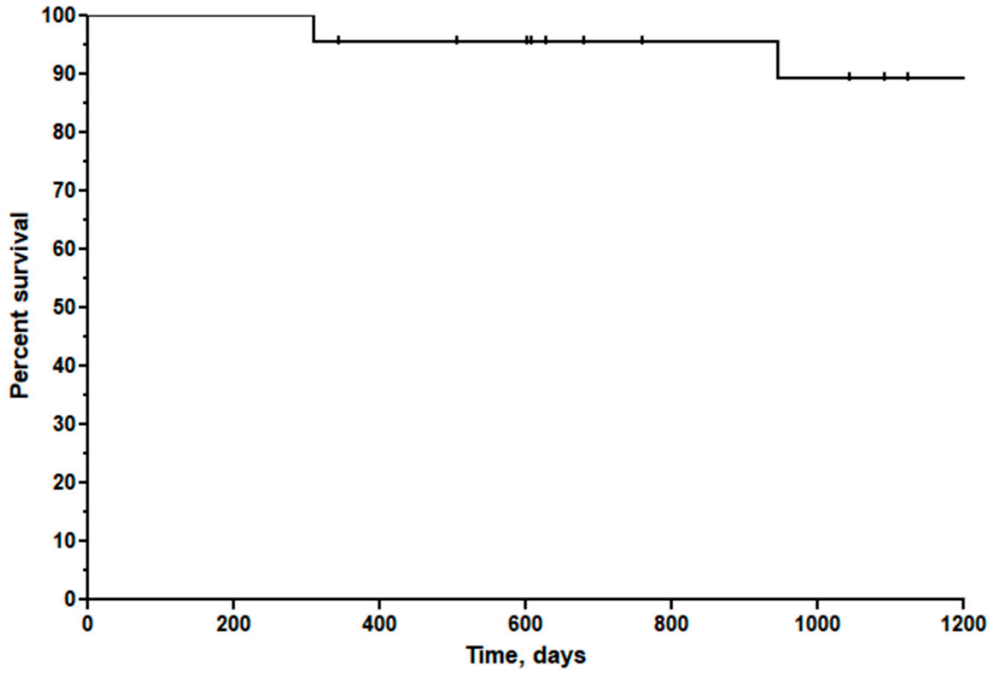

Figure S1: Kaplen-Meier plots corresponding to the data from Table S1. These plots were obtained based on Table S1 of Liu et al. (Liu et al., 2018). This Table has two columns, "OS" and "OS.time," that were used in GraphPad Prism software for survival curve analysis for BLCA (A), COAD (B), and UCS (C), indicating death/event as 1 and censored data as 0.
