# Supplementary material for: Assessing RNA-Seq Workflow Methodologies Using Shannon Entropy
Source: Biology (Basel). 2024 Jun 28;13(7):482. doi: 10.3390/biology13070482 (PMC11274087; doi:10.3390/biology13070482)
Supplement: Supplementary file 1 [file biology-13-00482-s001.zip › biology-3035206-supplementary/Figure S3.pdf]

A: STAD

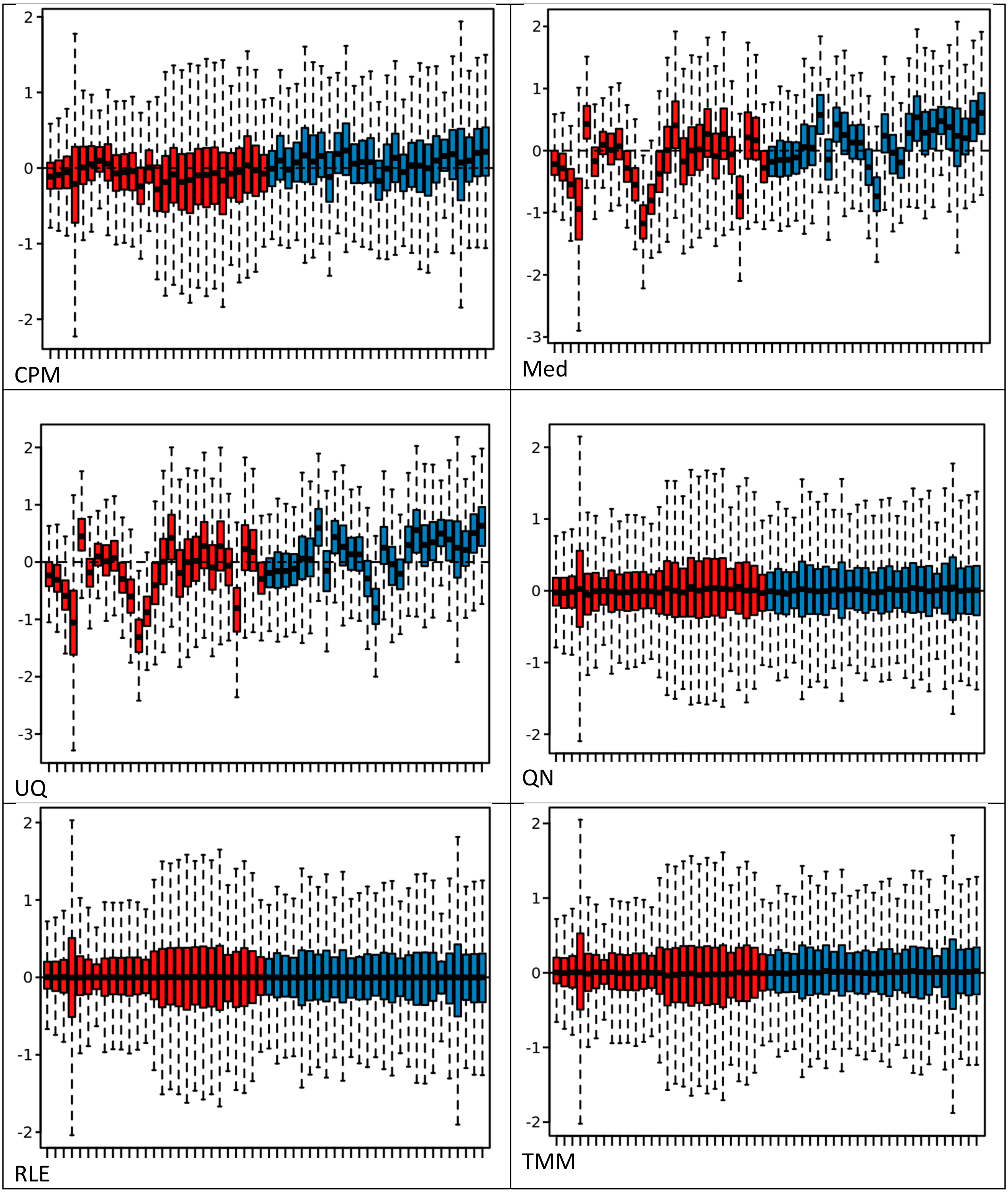

B: LUSC

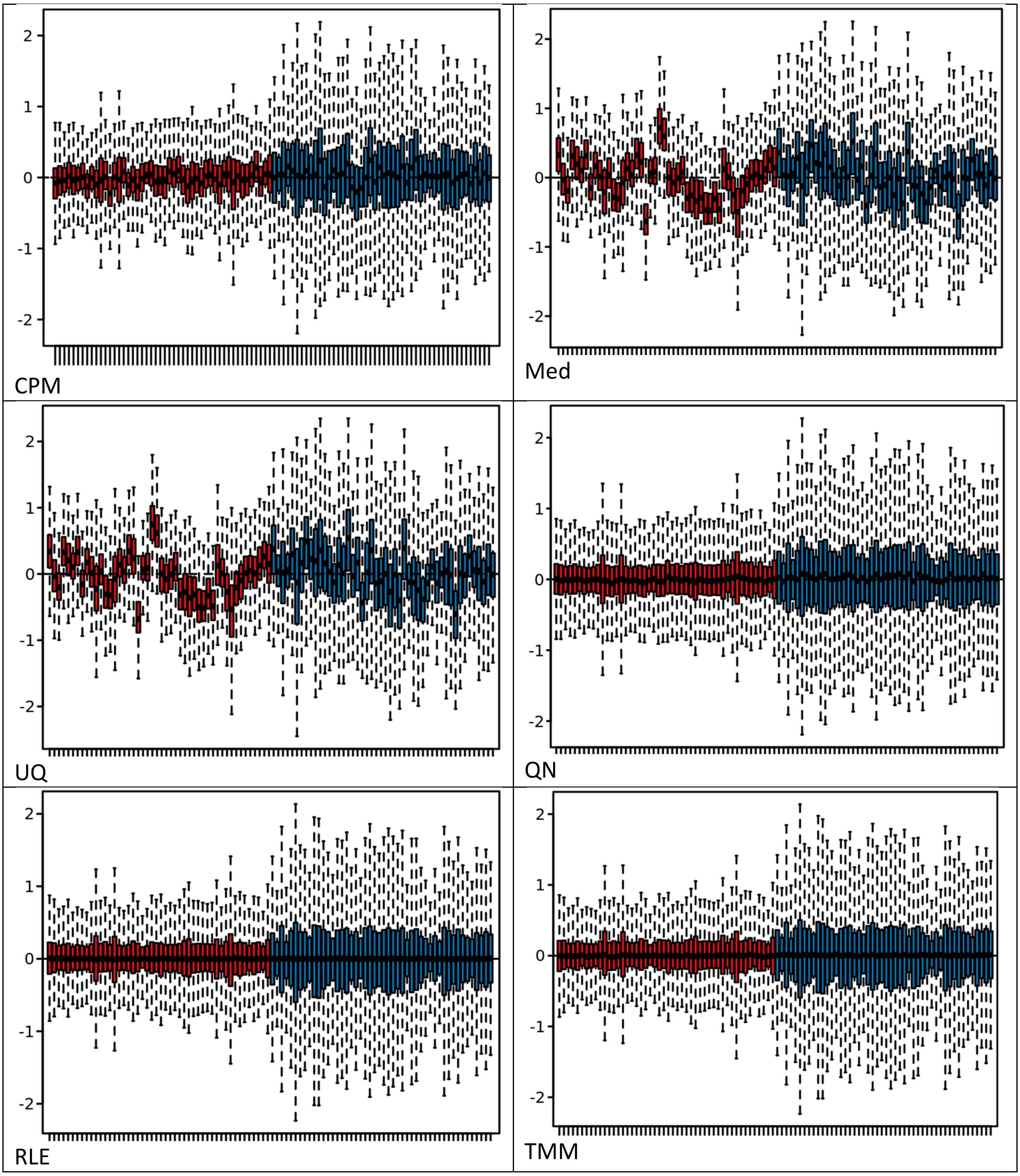

C: LIHC

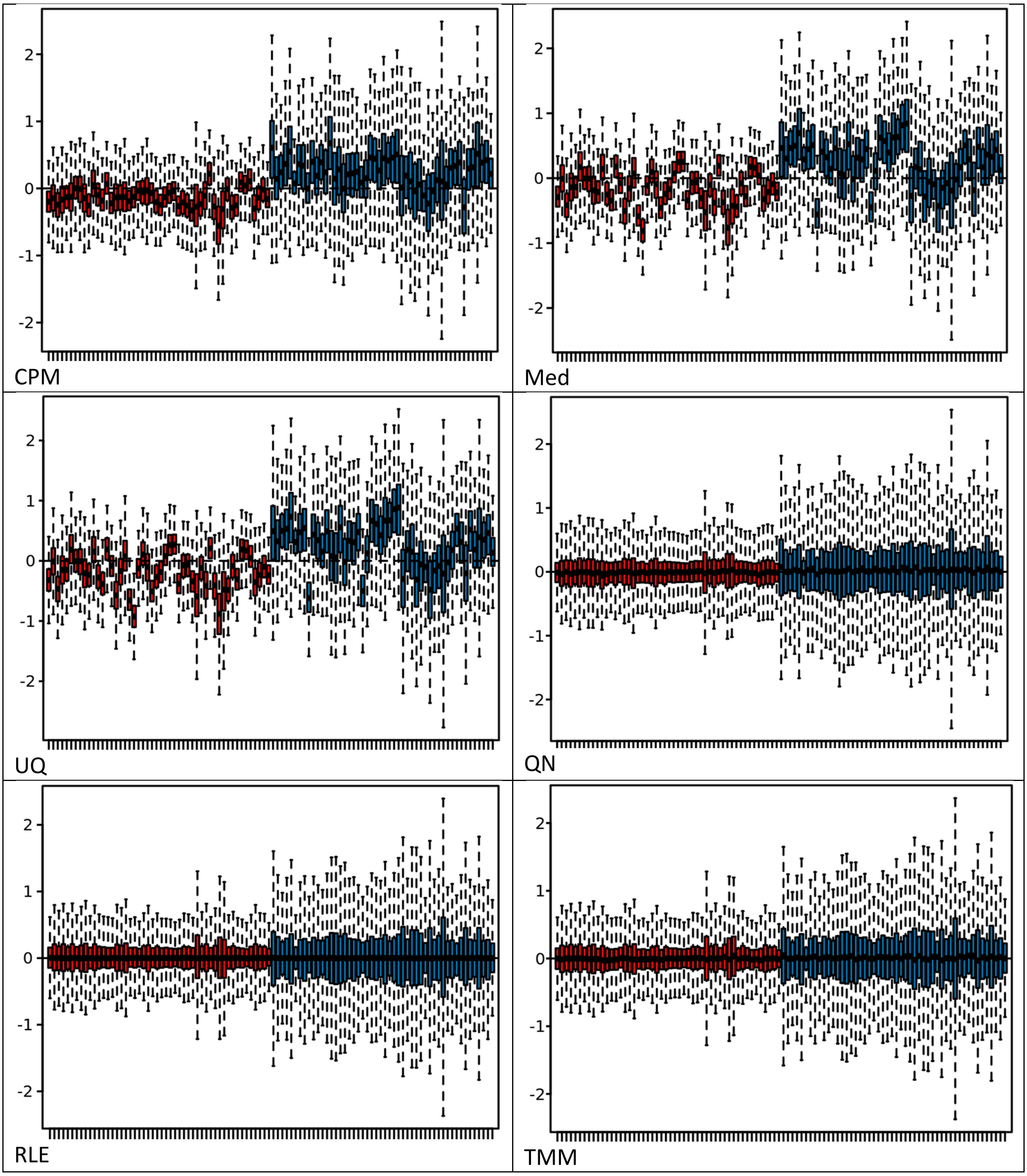

D: KIRC

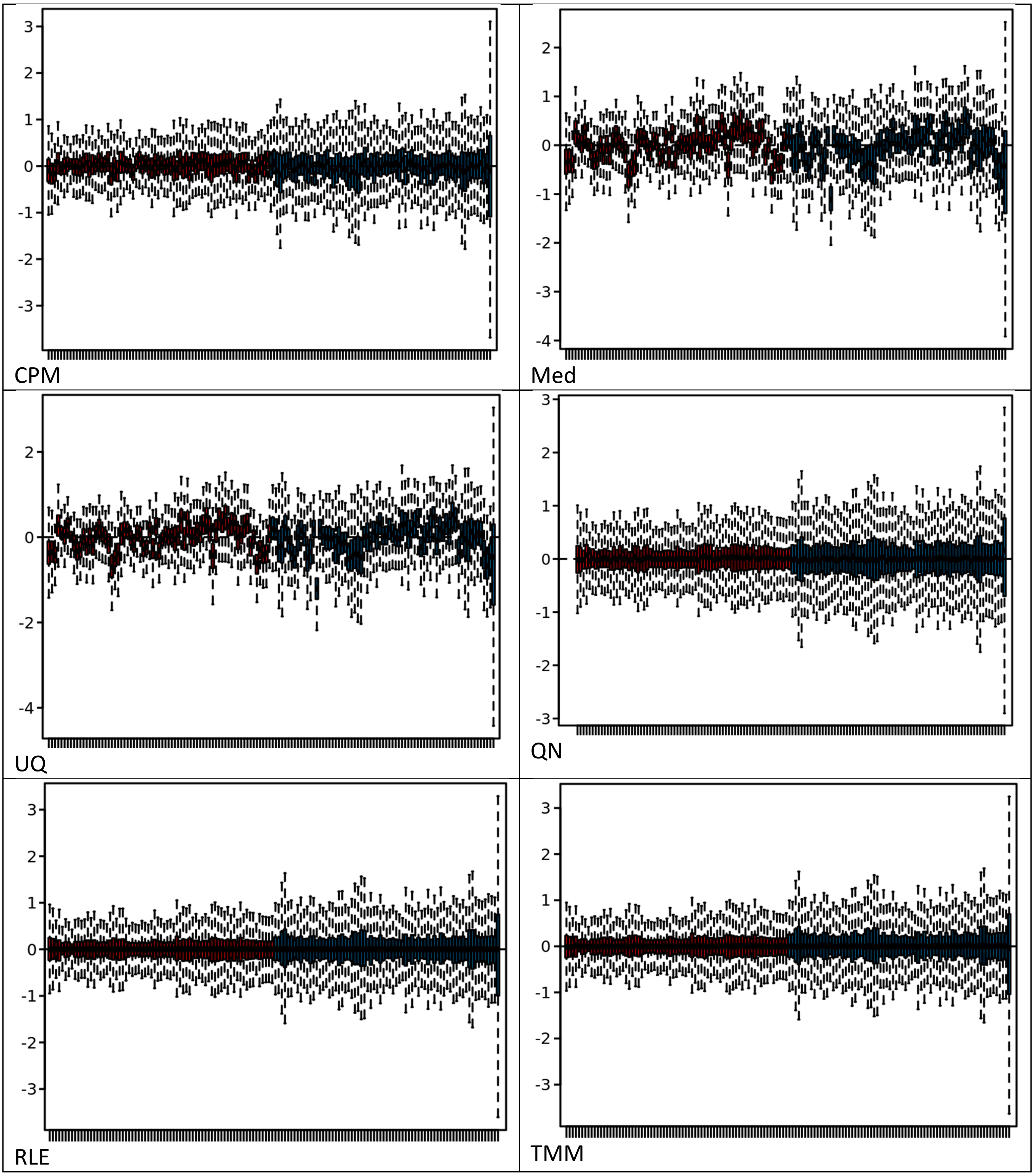

E: KIRP

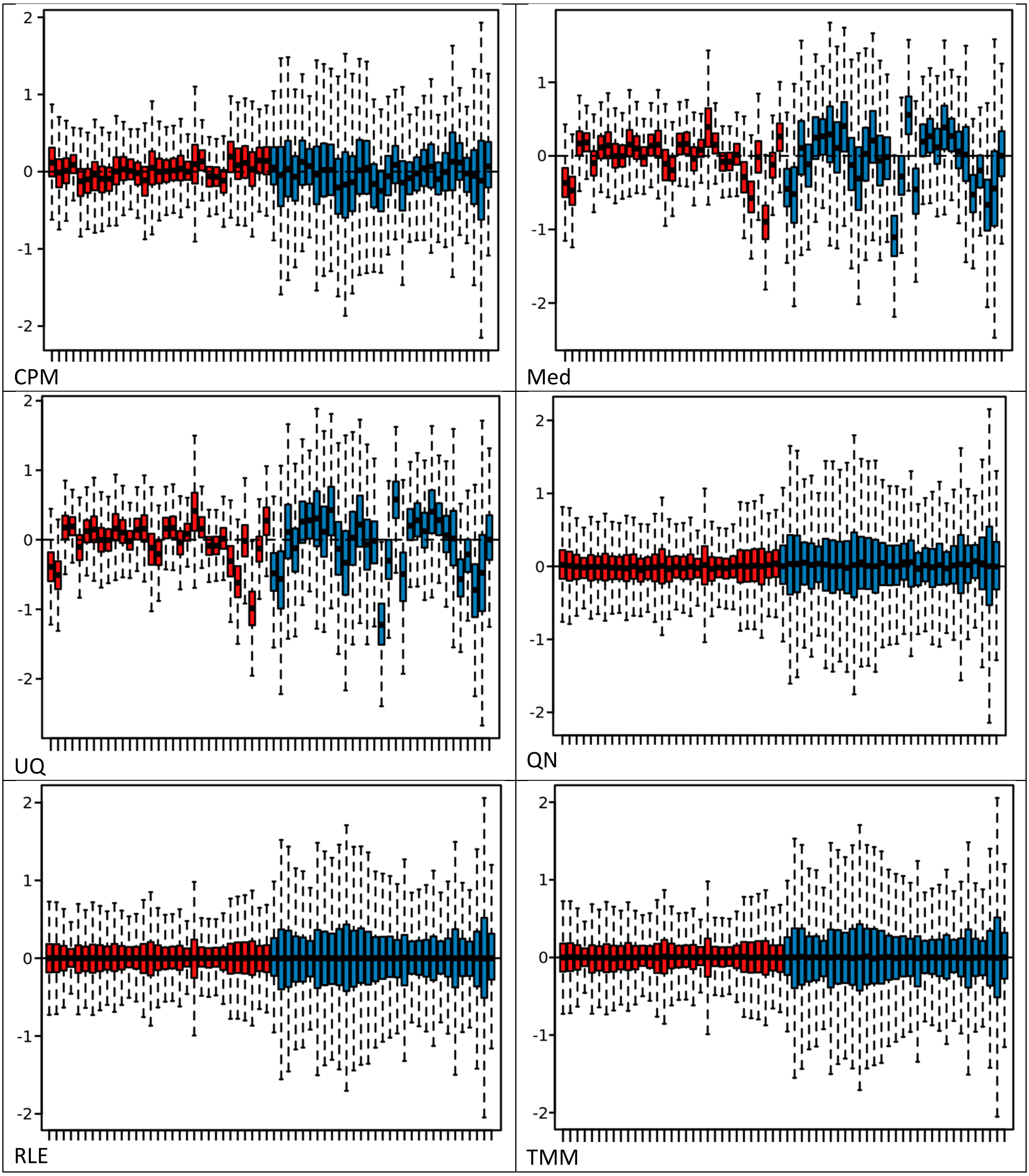

F: BRCA

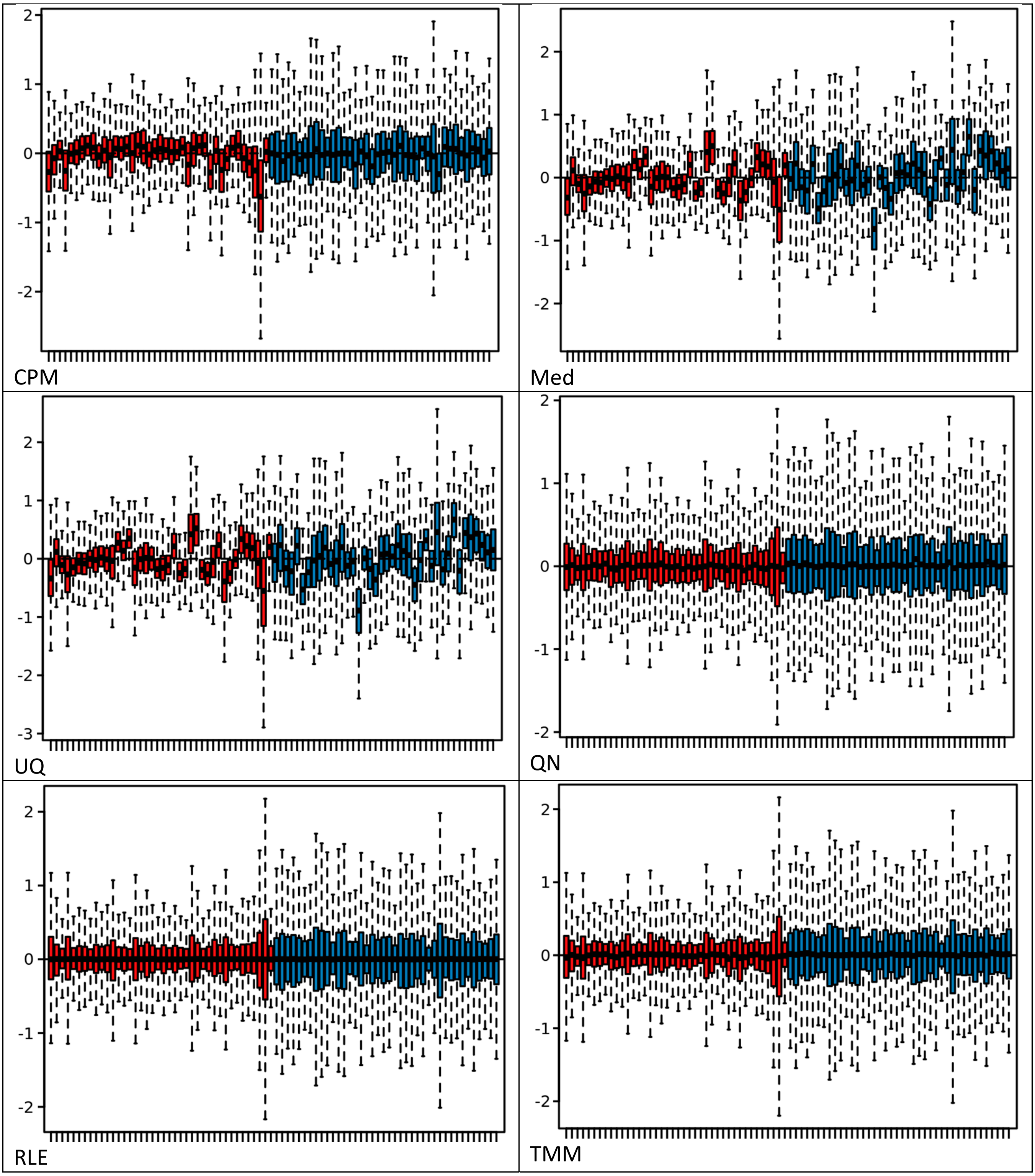

G: THCA

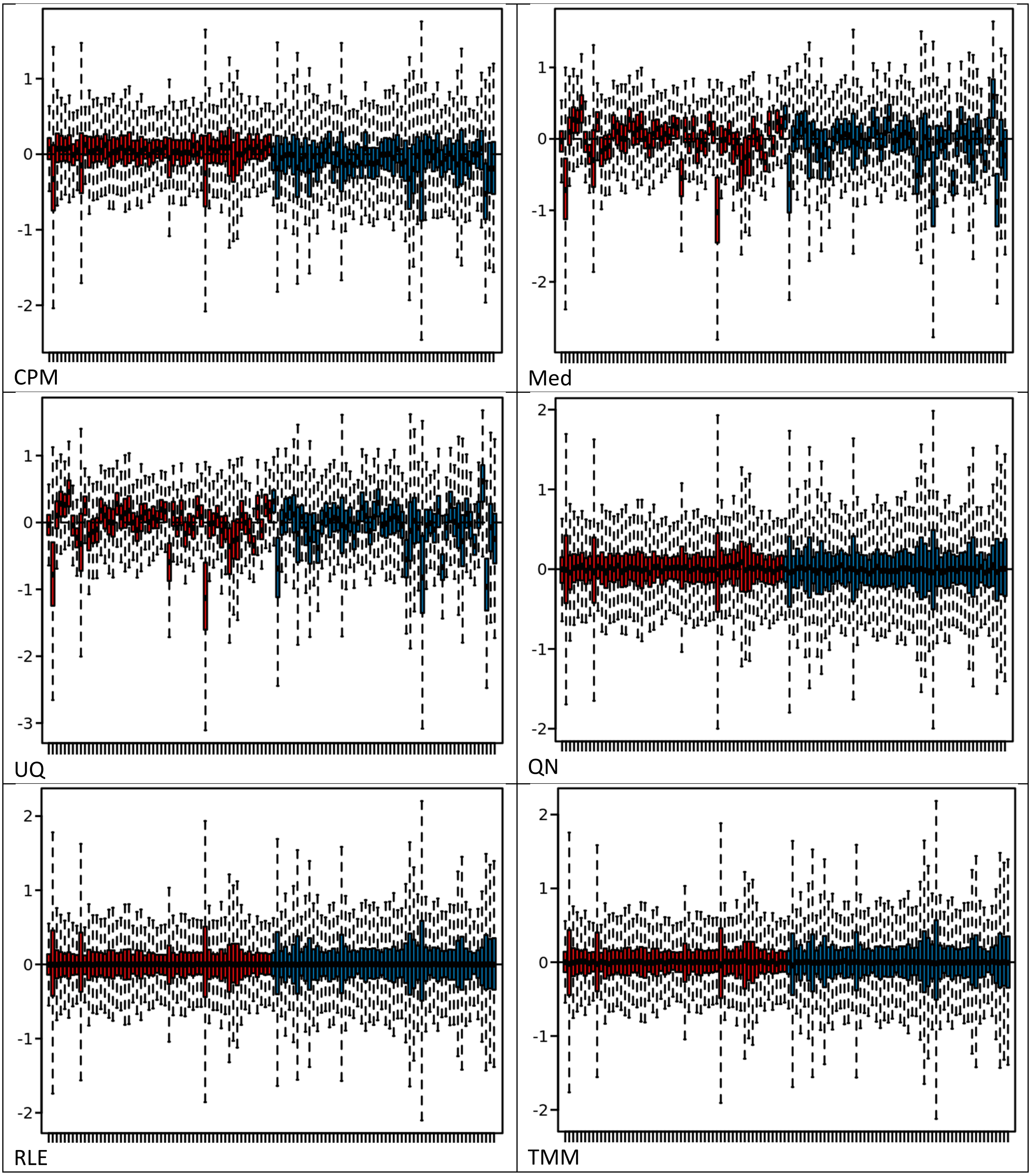

H: PRAD

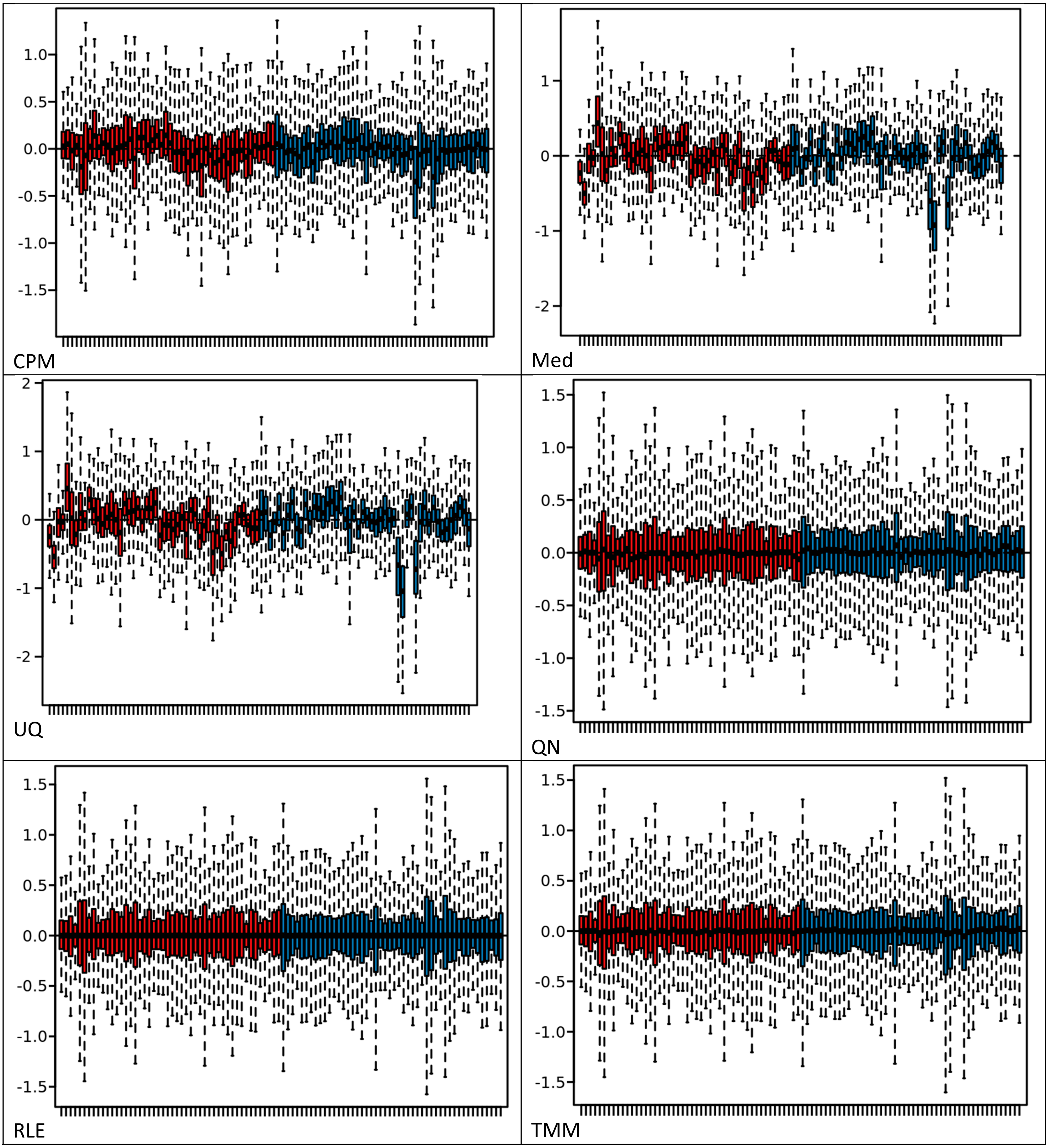

Figure S3: RLE plots produced by NormSeq for six normalzing method (UQ, Med, CPM, RLE, QN, and TMM) for STAD (A), LUSC (B), LIHC (C), KIRC (D), KIRP (E), BRCA (F), THCA (G), and PRAD (H). Cancer samples are plotted as blue and control as red.
