# Supplementary material for: Assessing RNA-Seq Workflow Methodologies Using Shannon Entropy
Source: Biology (Basel). 2024 Jun 28;13(7):482. doi: 10.3390/biology13070482 (PMC11274087; doi:10.3390/biology13070482)
Supplement: Supplementary file 1 [file biology-13-00482-s001.zip › biology-3035206-supplementary/Figure S4.pdf]

A: STAD

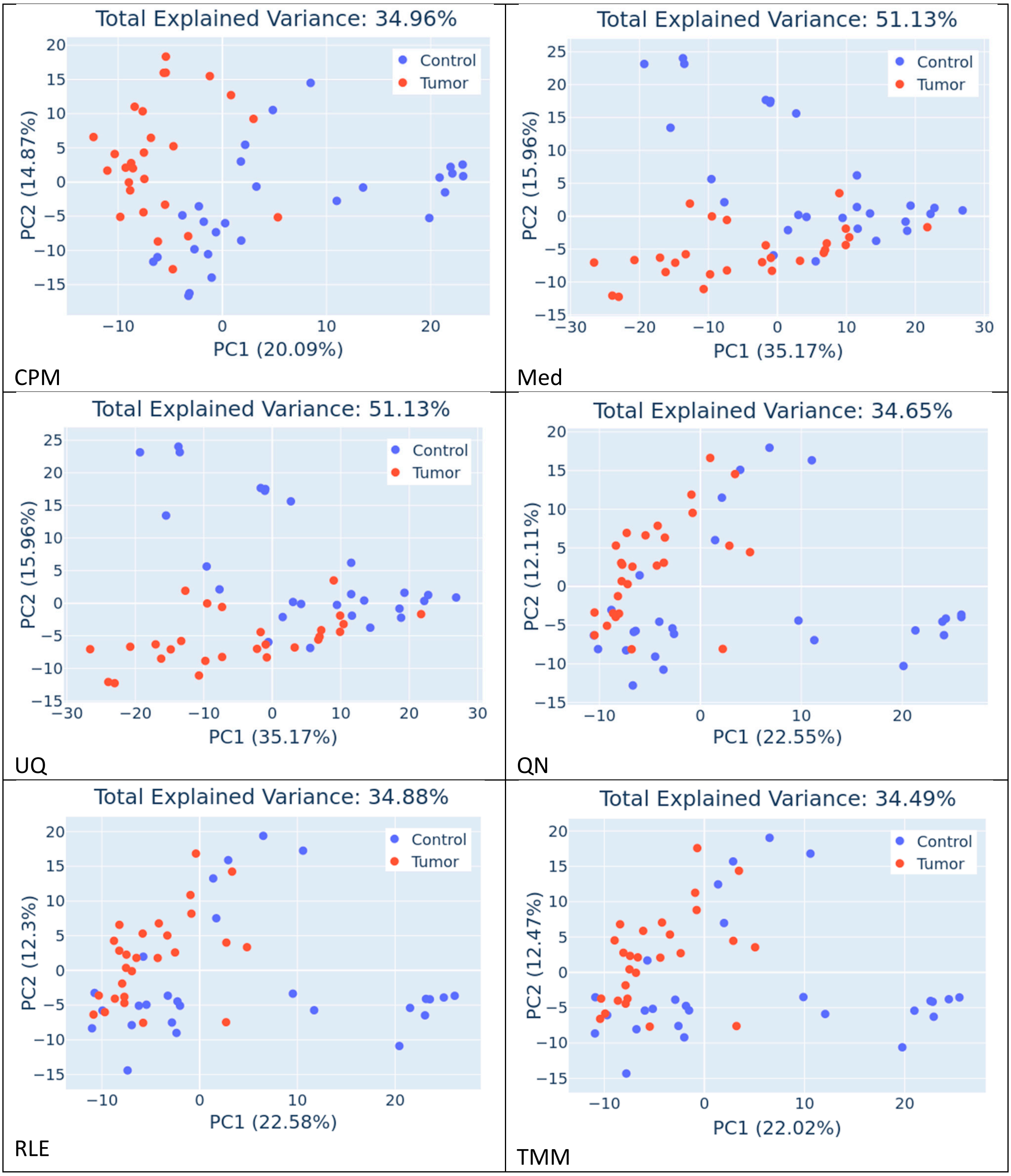
Total Explained Variance: 34.96%
Control
Tumor
PC2 (14.87%)
PC1 (20.09%)
CPM
Total Explained Variance: 51.13%
Control
Tumor
PC2 (15.96%)
PC1 (35.17%)
Med
Total Explained Variance: 51.13%
Control
Tumor
PC2 (15.96%)
PC1 (35.17%)
UQ
Total Explained Variance: 34.65%
PC2 (12.11%)
PC1 (22.55%)
QN
Total Explained Variance: 34.88%
Control
Tumor
PC2 (12.3%)
PC1 (22.58%)
RLE
Total Explained Variance: 34.49%
Control
Tumor
PC2 (12.47%)
PC1 (22.02%)
TMM

B: LUSC

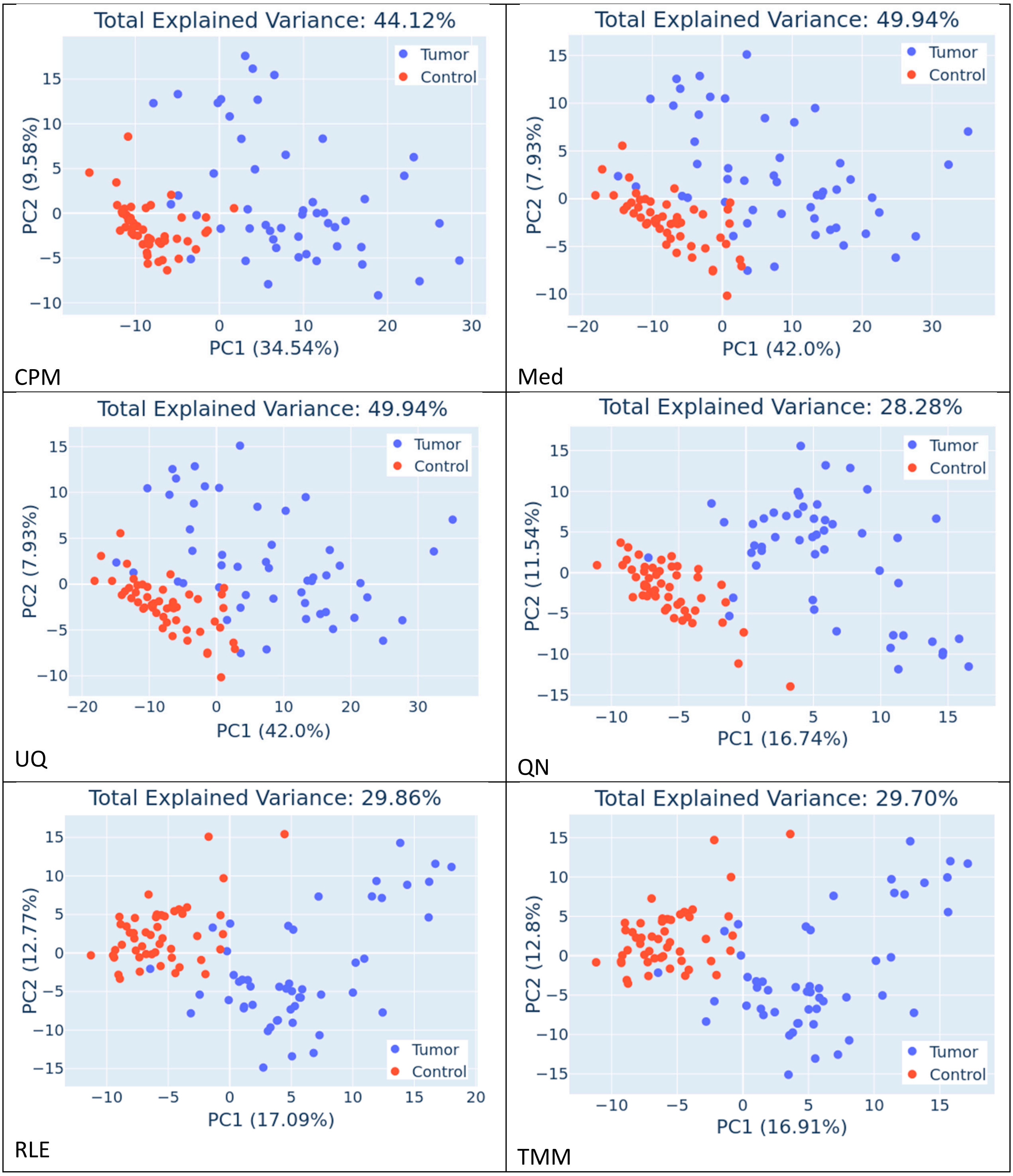
Total Explained Variance: 44.12%
PC2 (9.58%)
PC1 (34.54%)
Tumor
Control
CPM
Total Explained Variance: 49.94%
PC2 (7.93%)
PC1 (42.0%)
Tumor
Control
Med
Total Explained Variance: 49.94%
PC2 (7.93%)
PC1 (42.0%)
Tumor
Control
UQ
Total Explained Variance: 28.28%
PC2 (11.54%)
PC1 (16.74%)
Tumor
Control
QN
Total Explained Variance: 29.86%
PC2 (12.77%)
PC1 (17.09%)
Tumor
Control
RLE
Total Explained Variance: 29.70%
PC2 (12.8%)
PC1 (16.91%)
Tumor
Control
TMM

C: LIHC

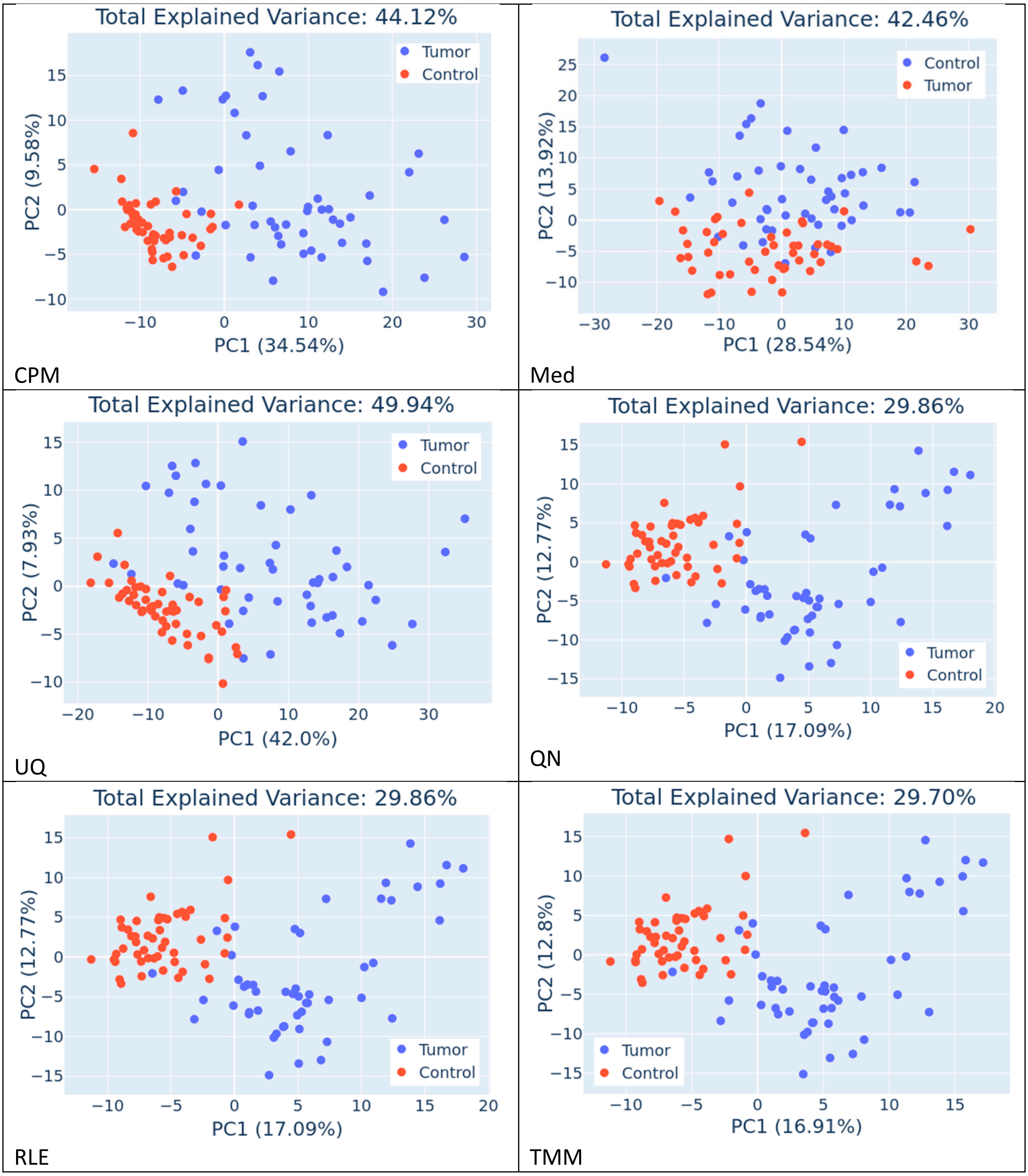
Total Explained Variance: 44.12%
Tumor
Control
PC2 (9.58%)
PC1 (34.54%)
CPM
Total Explained Variance: 42.46%
Control
Tumor
PC2 (13.92%)
PC1 (28.54%)
Med
Total Explained Variance: 49.94%
Tumor
Control
PC2 (7.93%)
PC1 (42.0%)
UQ
Total Explained Variance: 29.86%
Tumor
Control
PC2 (12.77%)
PC1 (17.09%)
QN
Total Explained Variance: 29.86%
Tumor
Control
PC2 (12.77%)
PC1 (17.09%)
RLE
Total Explained Variance: 29.70%
Tumor
Control
PC2 (12.8%)
PC1 (16.91%)
TMM

D: KIRC

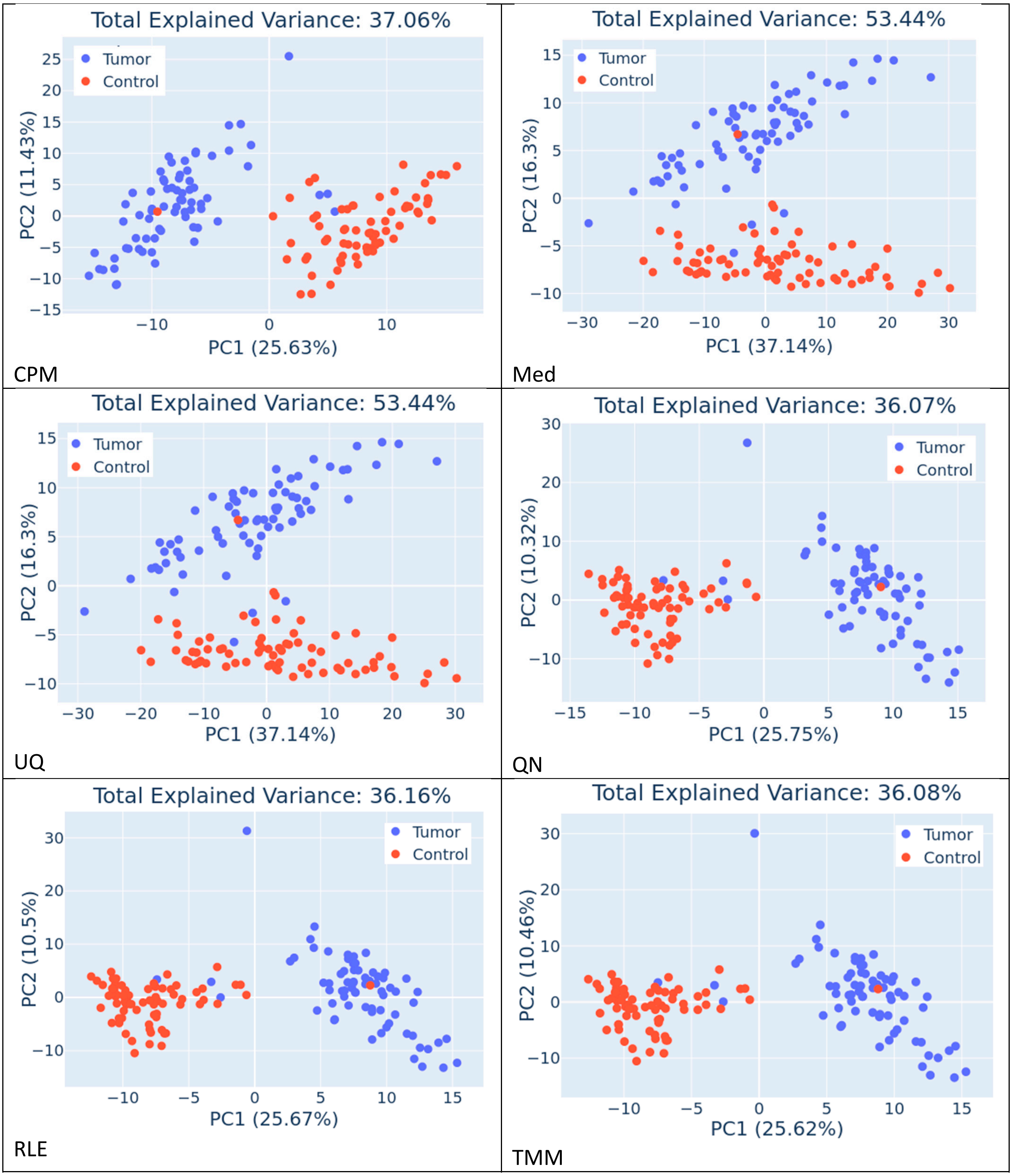
Total Explained Variance: 37.06%
Tumor
Control
PC2 (11.43%)
PC1 (25.63%)
CPM
Total Explained Variance: 53.44%
Tumor
Control
PC2 (16.3%)
PC1 (37.14%)
Med
Total Explained Variance: 53.44%
Tumor
Control
PC2 (16.3%)
PC1 (37.14%)
UQ
Total Explained Variance: 36.07%
Tumor
Control
PC2 (10.32%)
PC1 (25.75%)
QN
Total Explained Variance: 36.16%
Tumor
Control
PC2 (10.5%)
PC1 (25.67%)
RLE
Total Explained Variance: 36.08%
Tumor
Control
PC2 (10.46%)
PC1 (25.62%)
TMM

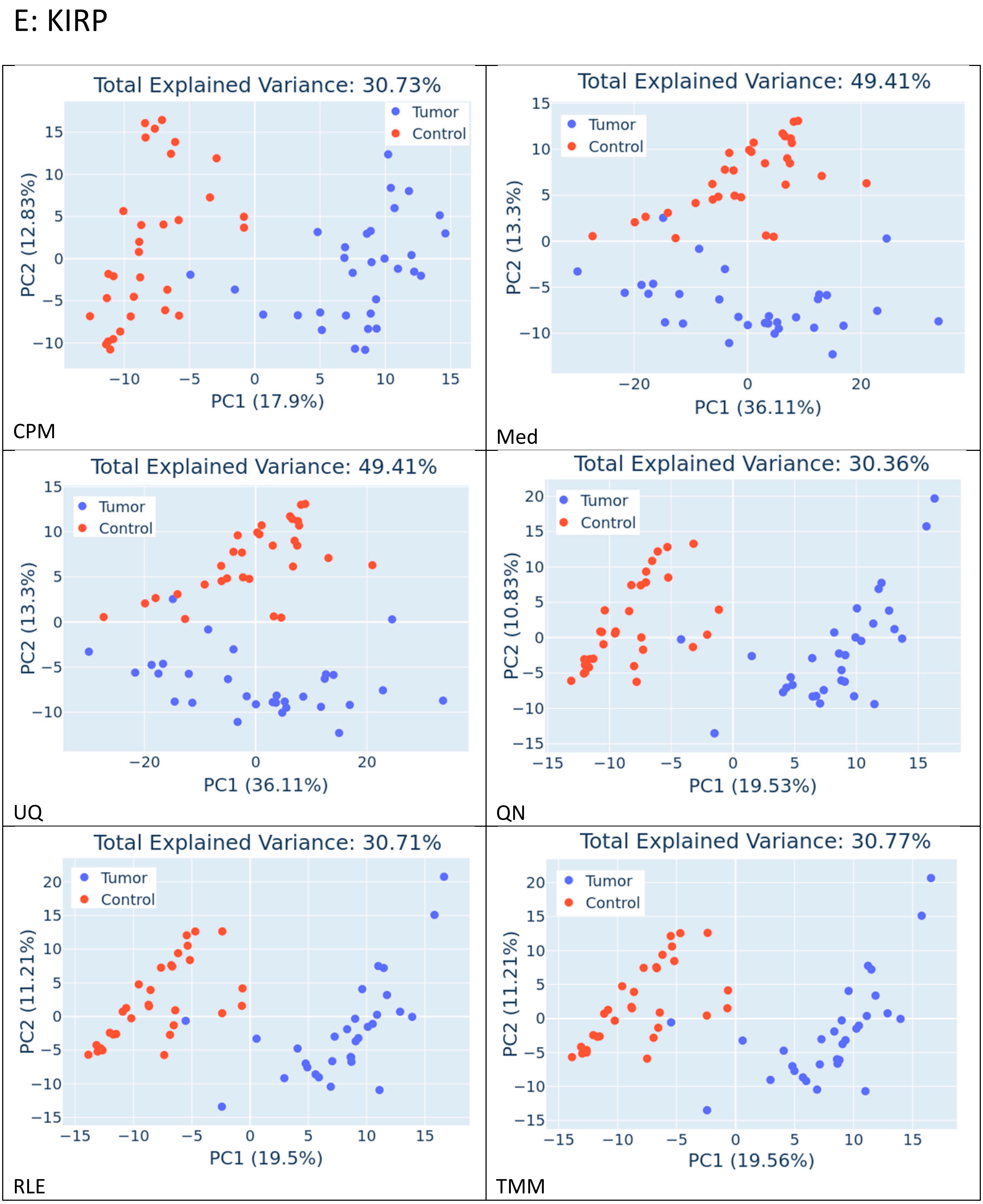

E: KIRP
Total Explained Variance: 30.73%
PC1 (17.9%)
PC2 (12.83%)
Tumor
Control
CPM
Total Explained Variance: 49.41%
PC1 (36.11%)
PC2 (13.3%)
Tumor
Control
Med
Total Explained Variance: 49.41%
PC1 (36.11%)
PC2 (13.3%)
Tumor
Control
UQ
Total Explained Variance: 30.36%
PC1 (19.53%)
PC2 (10.83%)
Tumor
Control
QN
Total Explained Variance: 30.71%
PC1 (19.5%)
PC2 (11.21%)
Tumor
Control
RLE
Total Explained Variance: 30.77%
PC1 (19.56%)
PC2 (11.21%)
Tumor
Control
TMM

F: BRCA

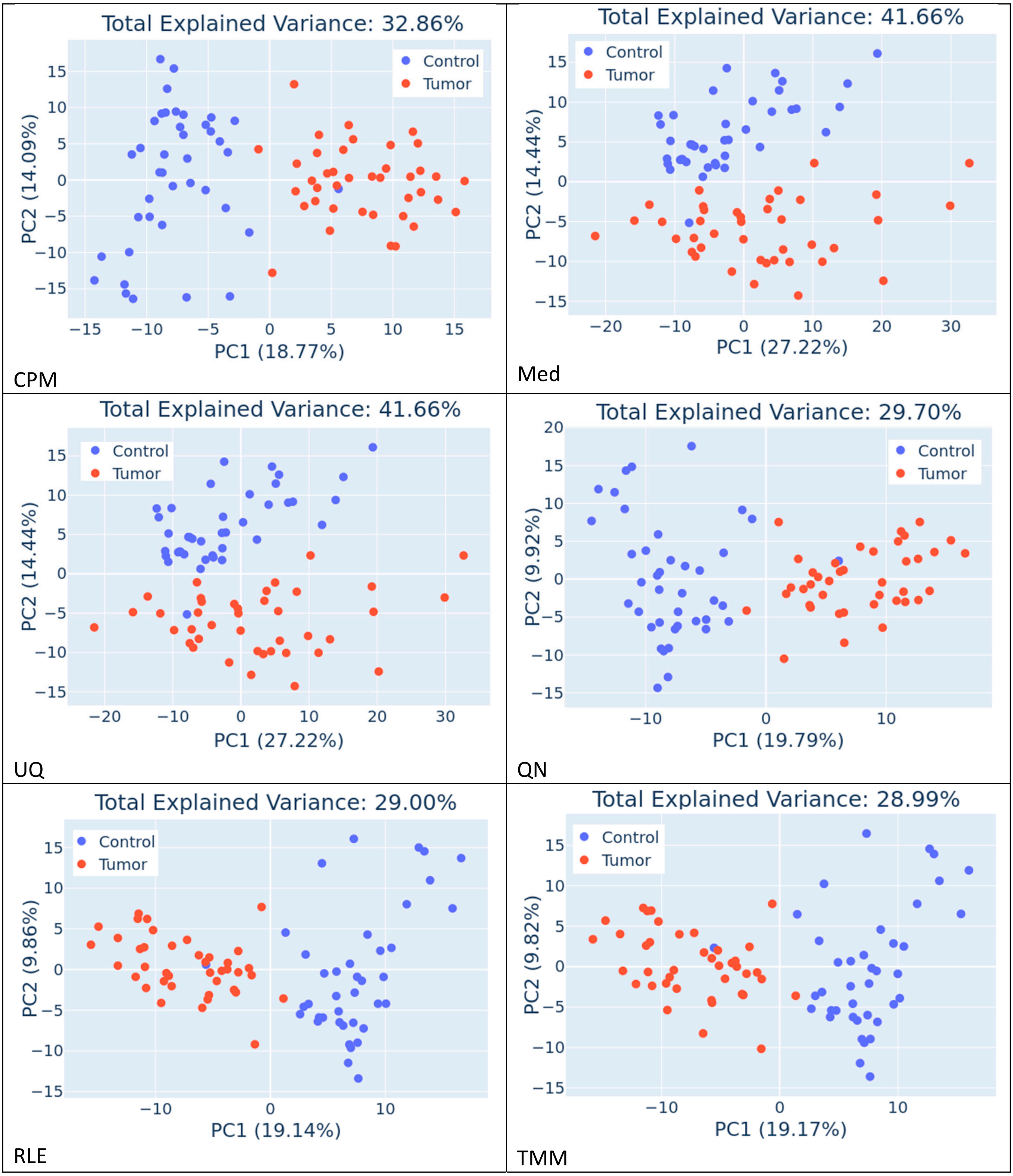
Total Explained Variance: 32.86%
Control
Tumor
PC2 (14.09%)
PC1 (18.77%)
CPM
Total Explained Variance: 41.66%
Control
Tumor
PC2 (14.44%)
PC1 (27.22%)
Med
Total Explained Variance: 41.66%
Control
Tumor
PC2 (14.44%)
PC1 (27.22%)
UQ
Total Explained Variance: 29.70%
Control
Tumor
PC2 (9.92%)
PC1 (19.79%)
QN
Total Explained Variance: 29.00%
Control
Tumor
PC2 (9.86%)
PC1 (19.14%)
RLE
Total Explained Variance: 28.99%
Control
Tumor
PC2 (9.82%)
PC1 (19.17%)
TMM

G: THCA

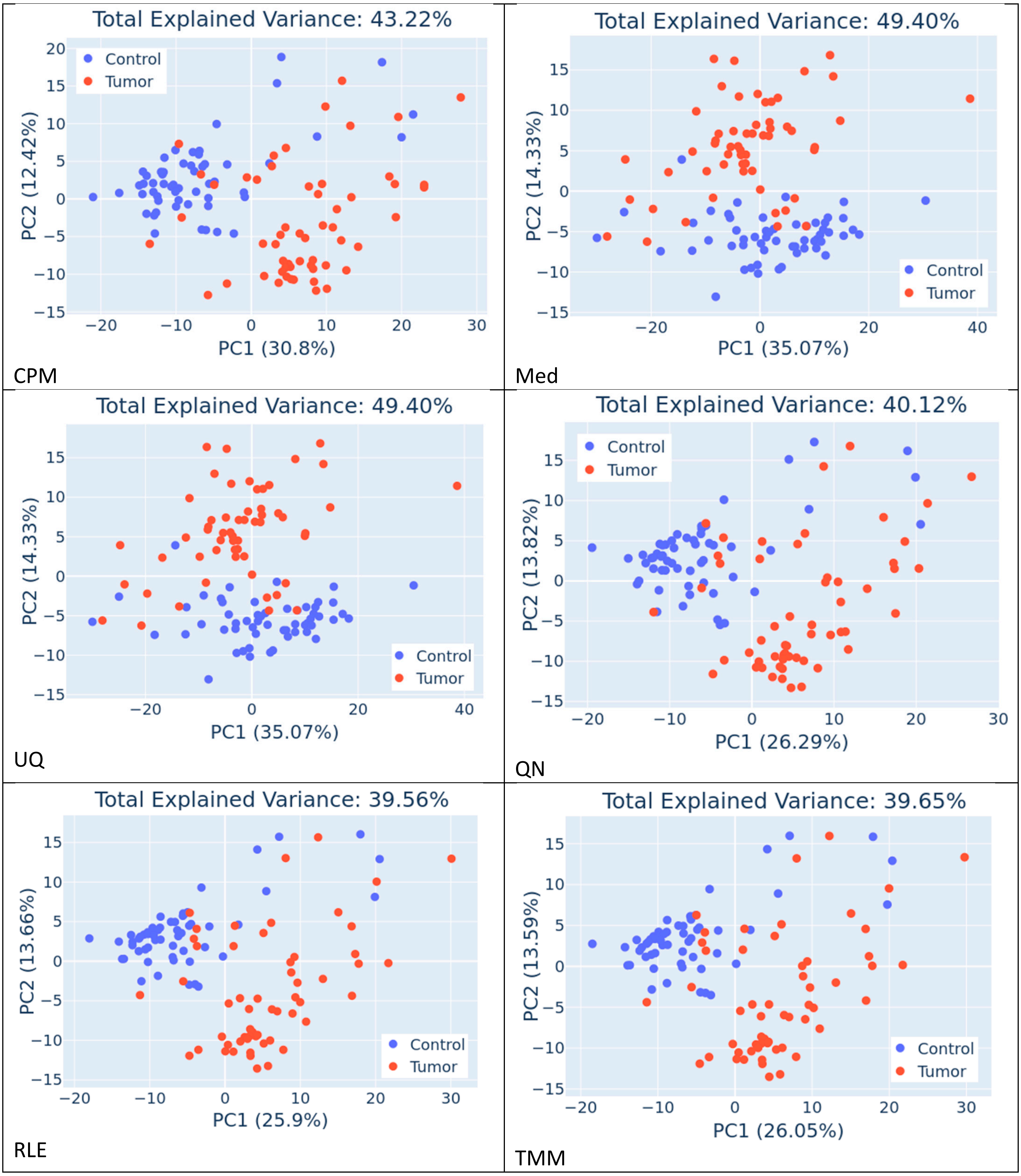
Total Explained Variance: 43.22%
Control
Tumor
PC2 (12.42%)
PC1 (30.8%)
CPM
Total Explained Variance: 49.40%
Control
Tumor
PC2 (14.33%)
PC1 (35.07%)
Med
Total Explained Variance: 49.40%
Control
Tumor
PC2 (14.33%)
PC1 (35.07%)
UQ
Total Explained Variance: 40.12%
Control
Tumor
PC2 (13.82%)
PC1 (26.29%)
QN
Total Explained Variance: 39.56%
Control
Tumor
PC2 (13.66%)
PC1 (25.9%)
RLE
Total Explained Variance: 39.65%
Control
Tumor
PC2 (13.59%)
PC1 (26.05%)
TMM

H: PRAD

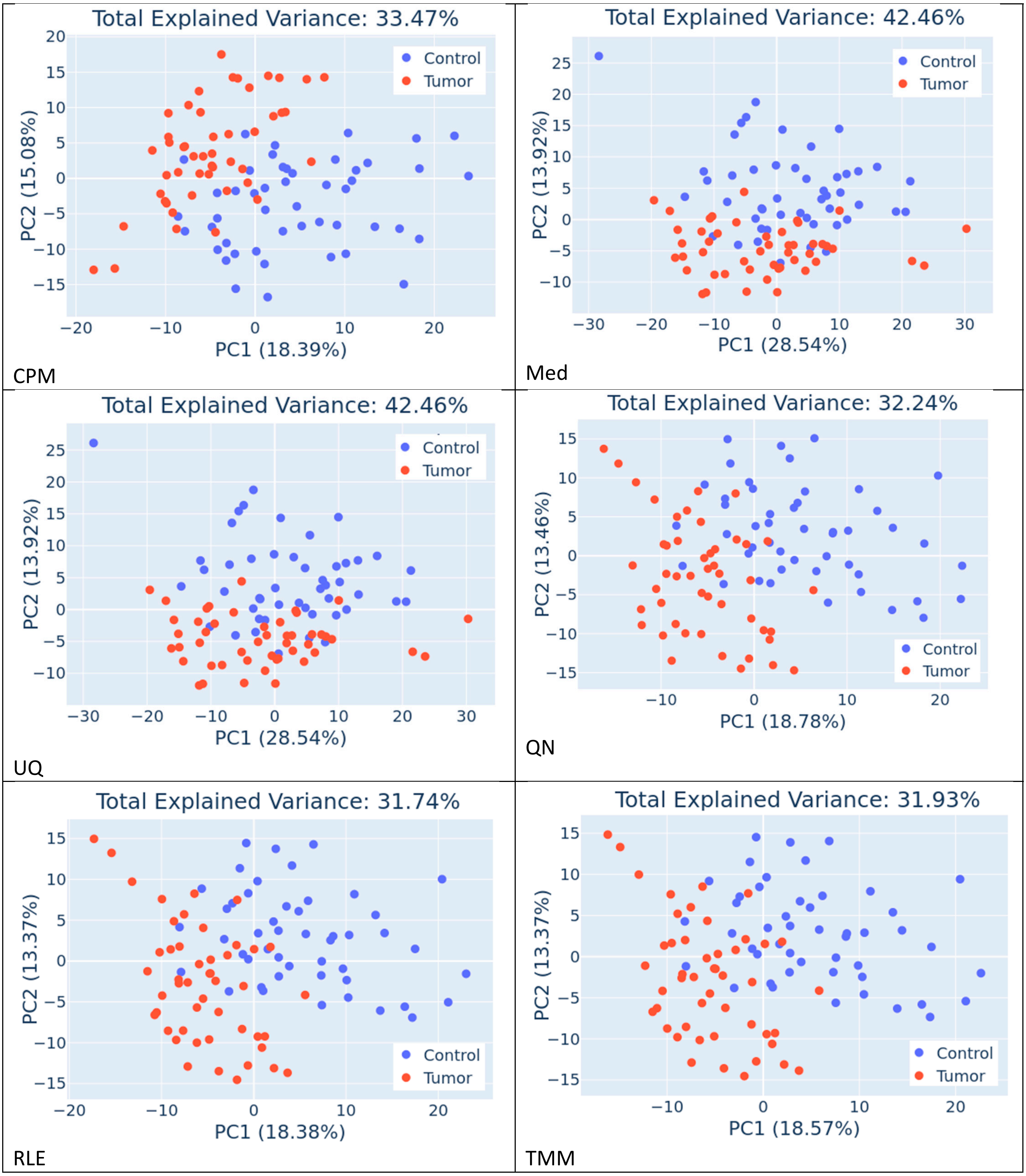
Total Explained Variance: 33.47%
PC1 (18.39%)
PC2 (15.08%)
Control
Tumor
CPM
Total Explained Variance: 42.46%
PC1 (28.54%)
PC2 (13.92%)
Control
Tumor
Med
Total Explained Variance: 42.46%
PC1 (28.54%)
PC2 (13.92%)
Control
Tumor
UQ
Total Explained Variance: 32.24%
PC1 (18.78%)
PC2 (13.46%)
Control
Tumor
QN
Total Explained Variance: 31.74%
PC1 (18.38%)
PC2 (13.37%)
Control
Tumor
RLE
Total Explained Variance: 31.93%
PC1 (18.57%)
PC2 (13.37%)
Control
Tumor
TMM

Figure S4: PCA plots produced by NormSeq for six normalzing method (UQ, Med, CPM, RLE, QN, and TMM) for STAD (A), LUSC (B), LIHC (C), KIRC (D), KIRP (E), BRCA (F), THCA (G), and PRAD (H).
