# Supplementary figures and images for: Assessing RNA-Seq Workflow Methodologies Using Shannon Entropy
Source: Biology (Basel). 2024 Jun 28;13(7):482. doi: 10.3390/biology13070482 (PMC11274087; doi:10.3390/biology13070482)

# DESeq2

## RLE plots

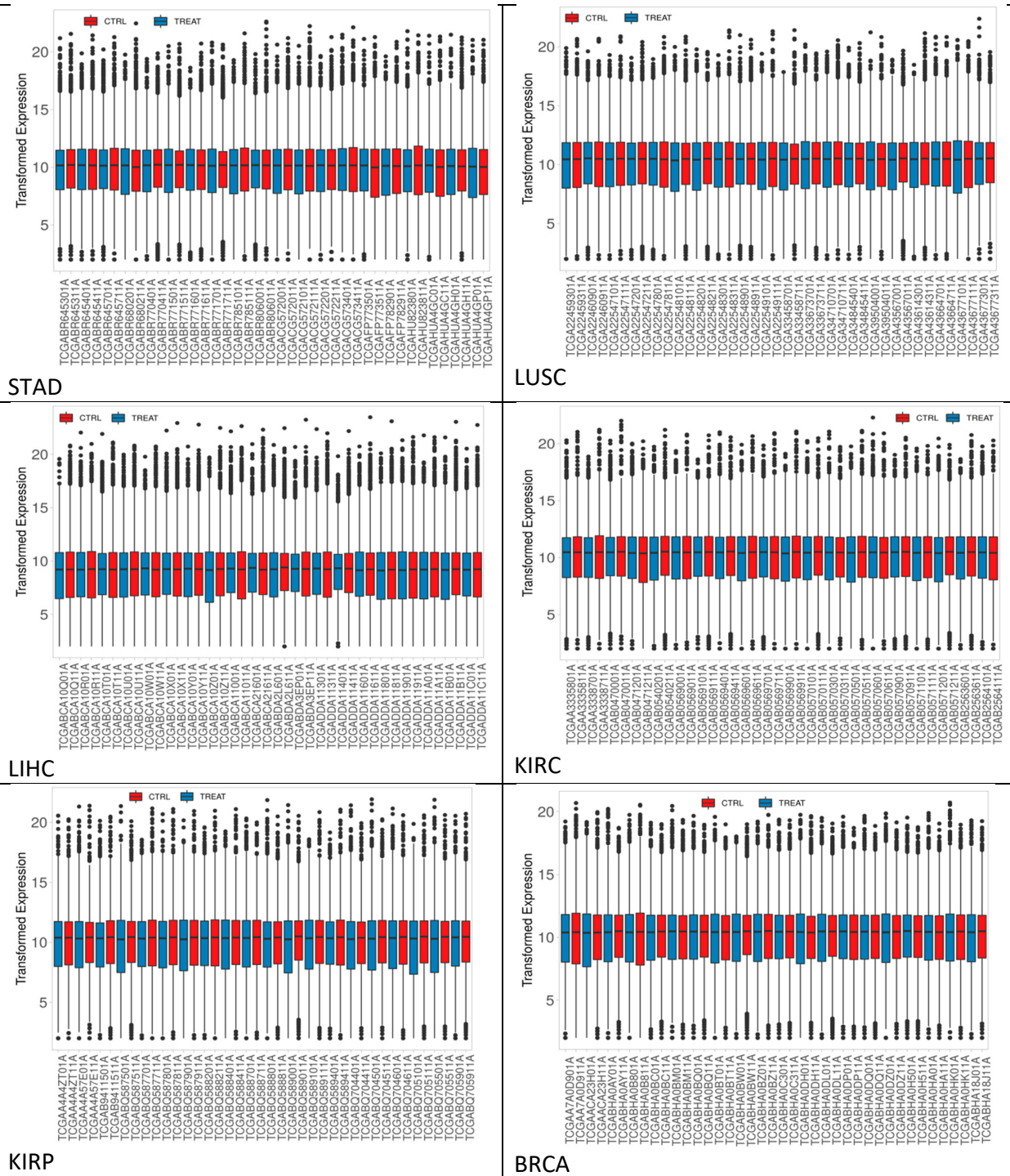

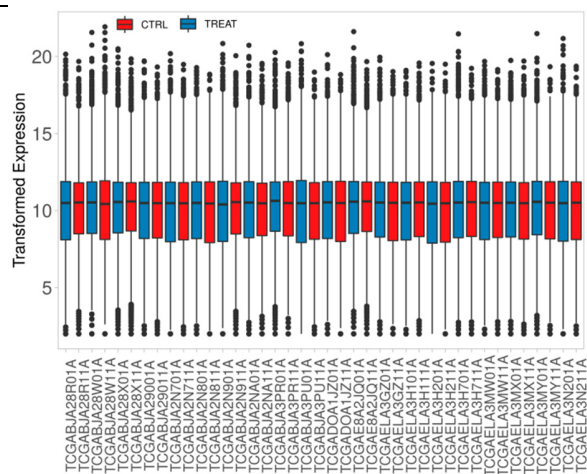

THCA

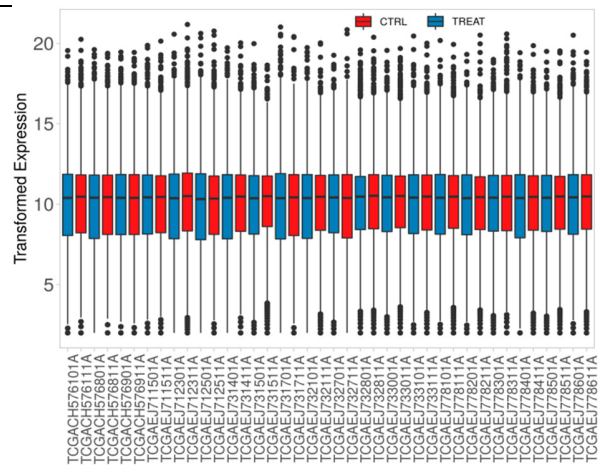

PRAD

## PCA plots

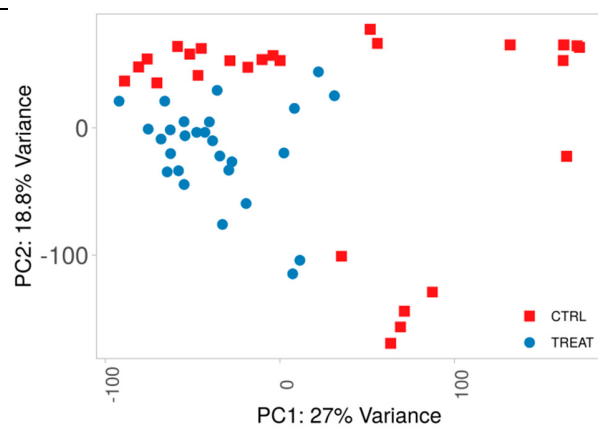

STAD

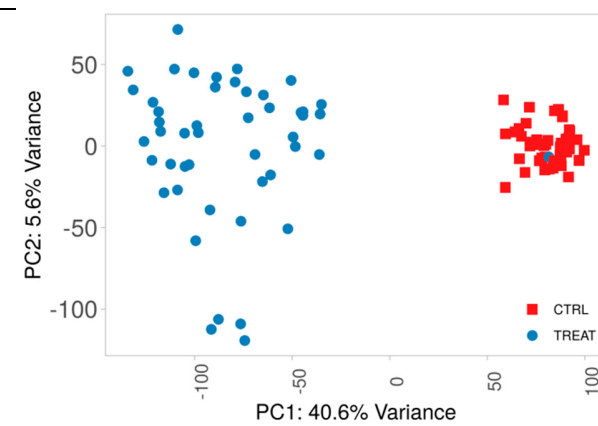

LUSC

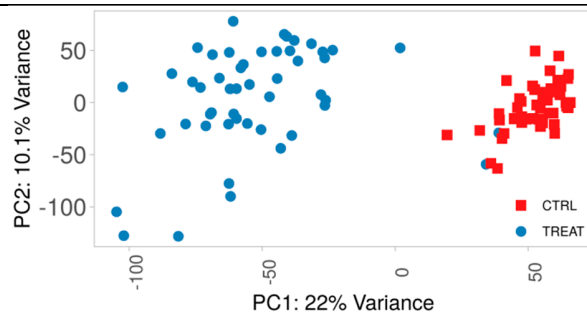

LIHC

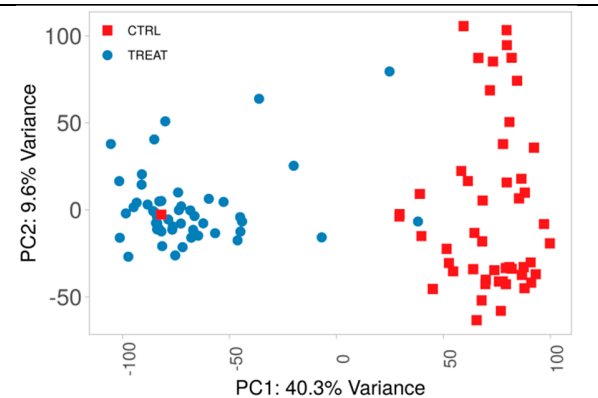

KIRC

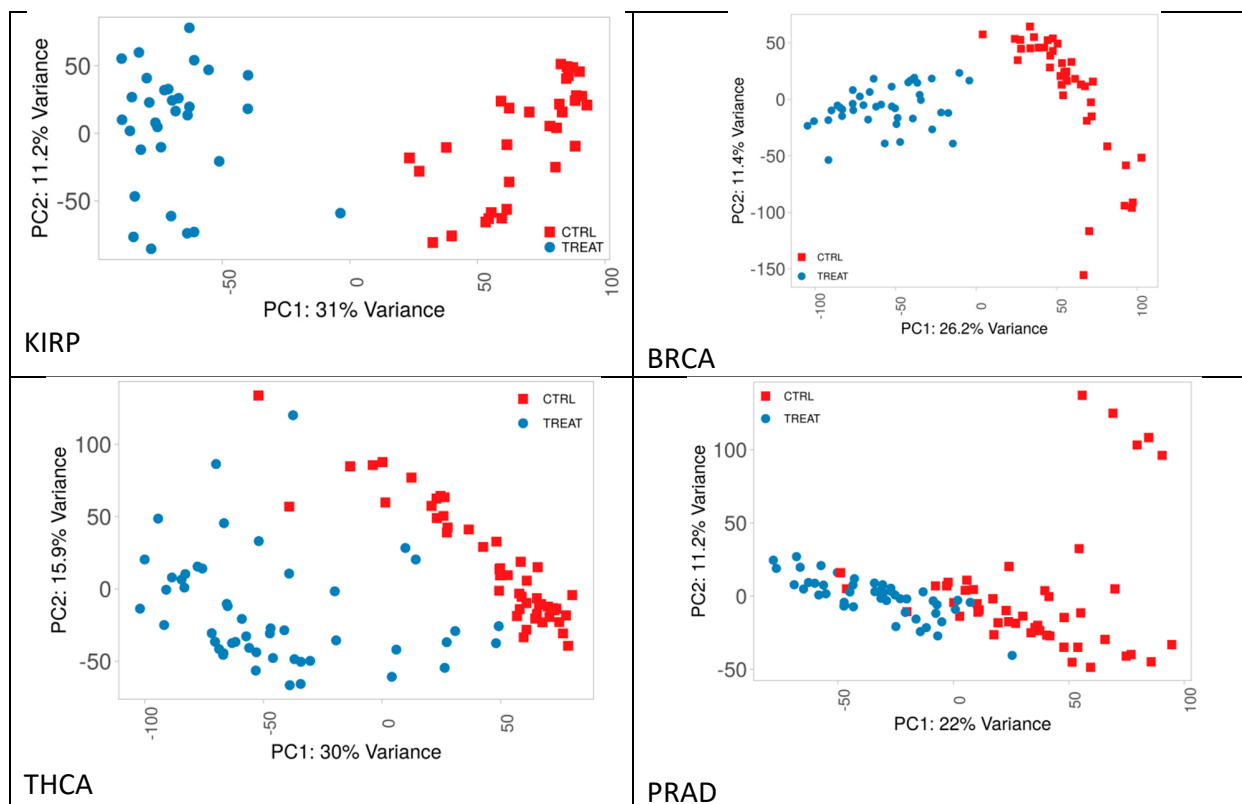

**Figure S2:** RLE and PCA plots of DESeq2.

Supplement: Supplementary file 1 [file biology-13-00482-s001.zip › biology-3035206-supplementary/Figure S2.pdf]
